# High rate of renal recovery in survivors of COVID-19 associated acute renal failure requiring renal replacement therapy

**DOI:** 10.1371/journal.pone.0244131

**Published:** 2020-12-28

**Authors:** Jacob S. Stevens, Kristen L. King, Shelief Y. Robbins-Juarez, Pascale Khairallah, Katherine Toma, Hector Alvarado Verduzco, Emily Daniel, Denzil Douglas, Andrew A. Moses, Yonatan Peleg, Piotr Starakiewicz, Miah T. Li, Daniel W. Kim, Kathleen Yu, Long Qian, Vaqar H. Shah, Max R. O'Donnell, Matthew J. Cummings, Jason Zucker, Karthik Natarajan, Adler Perotte, Demetra Tsapepas, Kiryluk Krzysztof, Geoffrey Dube, Eric Siddall, Shayan Shirazian, Thomas L. Nickolas, Maya K. Rao, Jonathan M. Barasch, Anthony M. Valeri, Jai Radhakrishnan, Ali G. Gharavi, S. Ali Husain, Sumit Mohan

**Affiliations:** 1 Division of Nephrology, Department of Medicine, Columbia University Irving Medical Center, New York, NY, United States of America; 2 Columbia University Renal Epidemiology Group, New York, NY, United States of America; 3 Vagelos College of Physicians and Surgeons, Columbia University, New York, NY, United States of America; 4 Division of Pulmonary, Allergy & Critical Care Medicine, Department of Medicine, Columbia University Irving Medical Center, New York, NY, United States of America; 5 Department of Epidemiology, Mailman School of Public Health, Columbia University, New York, NY, United States of America; 6 Division of Infectious Diseases, Department of Medicine, Columbia University Irving Medical Center, New York, NY, United States of America; 7 Department of Biomedical Informatics, Columbia University, New York, NY, United States of America; 8 Department of Pharmacy, New-York Presbyterian Hospital, New York, NY, United States of America; 9 Department of Quality, New-York Presbyterian Hospital, New York, NY, United States of America; Universidade do Extremo Sul Catarinense, BRAZIL

## Abstract

**Introduction:**

A large proportion of patients with COVID-19 develop acute kidney injury (AKI). While the most severe of these cases require renal replacement therapy (RRT), little is known about their clinical course.

**Methods:**

We describe the clinical characteristics of COVID-19 patients in the ICU with AKI requiring RRT at an academic medical center in New York City and followed patients for outcomes of death and renal recovery using time-to-event analyses.

**Results:**

Our cohort of 115 patients represented 23% of all ICU admissions at our center, with a peak prevalence of 29%. Patients were followed for a median of 29 days (2542 total patient-RRT-days; median 54 days for survivors). Mechanical ventilation and vasopressor use were common (99% and 84%, respectively), and the median Sequential Organ Function Assessment (SOFA) score was 14. By the end of follow-up 51% died, 41% recovered kidney function (84% of survivors), and 8% still needed RRT (survival probability at 60 days: 0.46 [95% CI: 0.36–0.56])). In an adjusted Cox model, coronary artery disease and chronic obstructive pulmonary disease were associated with increased mortality (HRs: 3.99 [95% CI 1.46–10.90] and 3.10 [95% CI 1.25–7.66]) as were angiotensin-converting-enzyme inhibitors (HR 2.33 [95% CI 1.21–4.47]) and a SOFA score >15 (HR 3.46 [95% CI 1.65–7.25).

**Conclusions and relevance:**

Our analysis demonstrates the high prevalence of AKI requiring RRT among critically ill patients with COVID-19 and is associated with a high mortality, however, the rate of renal recovery is high among survivors and should inform shared-decision making.

## Introduction

The emergence of severe acute respiratory syndrome-associated coronavirus 2 (SARS-CoV-2) as a novel pathogen causing COVID-19 with global infection rates on a pandemic scale has taken many lives and stressed healthcare systems. While the recognition of acute respiratory distress syndrome (ARDS) and the obligate need for resource planning for ventilatory support was widespread, the extent of severe acute kidney injury (AKI) necessitating renal replacement therapy (RRT) and the associated prognosis for these individuals remains poorly defined given the lack of adequate phenotyping and the limited follow up in early reports.

Among patients with ARDS from all causes, 24–44% develop AKI [1,2], including 10–14% who develop AKI severe enough to require RRT [3]. AKI is an independent predictor of mortality among patients with ARDS of all causes [4] and appears to be associated with poor outcomes among patients with COVID-19 [5,6]. However, there remains uncertainty about this effect given that early cohort descriptions often did not include *any* kidney outcomes or RRT requirements in their ICU cohorts [7–9], and those that did indicated a wide range of incident AKI of any severity: 2–37% for all hospitalized patients [5,6,10–14], 8–75% among critically ill patients, and 5–31% with AKI requiring RRT in the ICU [12,15–21].

Early in the course of the pandemic, New York City became the disease epicenter in the United States. A surge of critically ill patients with COVID-19 requiring intensive care, created unprecedented challenges for resources while raising important questions about the clinical course and prognosis for these patients. Additionally, understanding the burden of complications, particularly those that are associated with intense resource needs such as RRT is critical for resource planning, especially as hospitals experience acute, critical shortages of related supplies, equipment, and personnel. Providers, patients, and their families who are faced with important clinical decisions about the care of these patients need to be better informed about the prognosis (both mortality and kidney recovery) of these patients in order to participate in shared-decision. We describe the detailed clinical phenotype and outcomes of mortality and kidney recovery of 115 critically ill patients infected with SARS-CoV-2 who developed severe AKI requiring RRT from a large, quaternary care academic hospital in NYC.

## Methods

### Cohort identification

We retrospectively identified all patients with a positive test result for SARS-CoV-2 from either a nasopharyngeal or oropharyngeal swab PCR who were admitted to an ICU and received RRT (defined as hemodialysis, continuous renal replacement therapy (CRRT), or acute peritoneal dialysis) for AKI between March 1st and April 18th, 2020 at New York Presbyterian-Columbia University Irving Medical Center and The Allen Pavilion. Patients with end stage kidney disease (ESKD) on RRT prior to admission were excluded from the study. This study protocol was approved by the Institutional Review Board of Columbia University Irving Medical Center and the requirement for informed consent was waived.

### Clinical characteristics and outcomes

Details of the patients’ hospital course, demographics, clinical data, laboratory data and clinical outcomes were obtained using a combination of manual chart review of the electronic medical record and laboratory data extraction from the clinical data warehouse (charts accessed up until June 7, 2020). Baseline serum creatinine (SCr) was determined by using the median SCr from 365 to 7 days before presentation when available or by the nadir in the 7 days before presentation. If neither were available, then the patient was considered to have an unknown baseline (70% of patients). Medical records were queried for orders for RRT and patients with ESRD were excluded in order to define our cohort of patients with severe AKI (defined as KDIGO Stage 3 AKI requiring RRT). Sequential organ function assessment (SOFA) scores [22] were calculated for the first 24 hours of ICU admission and again for the 24 hours prior to RRT initiation. For the neurologic component, the Glasgow coma scale (GCS) score was used when available, but in the 35 patients without a documented GCS in those timeframes, their Richmond Agitation-Sedation Score was converted into an equivalent SOFA neurologic component score [23]. Clinical data were insufficient to calculate SOFA scores for 14 patients (12%).

Laboratory values were obtained for two specific time points during hospitalization: the first value that was obtained within 24 hours following ICU admission, and the most recent value in the 24-hour window preceding RRT initiation. Minimum and maximum laboratory values were calculated from all values across the patient's hospital admission. Laboratory results reported to be above or below the limit of detection were converted into a continuous value one unit above or below the threshold (S1 Table).

Patients were classified into three groups based on the outcome at the end of follow up: (1) death, (2) recovery of kidney function (off RRT at the end of thefollow-up), or (3) RRT still indicated. For mortality outcome assessments, patients were censored on hospital discharge or at the end of study follow-up (June 7, 2020).

### Statistical analysis

Descriptive statistics were calculated for patient demographics, comorbidities, medications, COVID-19 disease presentation, laboratory values, and hospital course for each patient event group and are presented as median (interquartile range) for continuous values or counts (column percentage) for categorical values.

Patient mortality from the time of RRT initiation was examined using Kaplan-Meier curves, as well as unadjusted and adjusted Cox regression models. Patient demographic characteristics, past medical history, medications, selected laboratory and clinical parameters including urine output, and SOFA score tertiles as a measure of disease severity were examined in bivariable analyses and considered for inclusion in the multivariable model based on clinical judgement and evidence of effect in the univariable analyses. An alpha of 0.05 was considered statistically significant. Analyses were conducted in Stata/MP 15.1 (StataCorp, College Station, TX).

## Results

**Fig 1:**
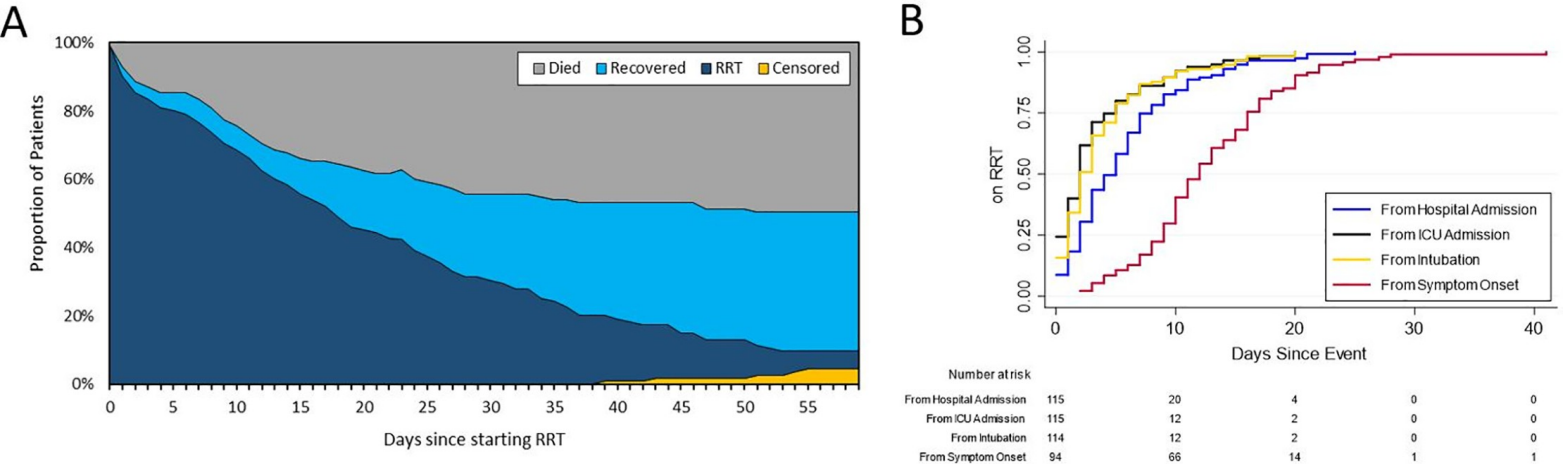
Outcomes of patients receiving renal replacement therapy for acute kidney injury. (A) Relative composition of the cohort showing the proportion of patients who have died (gray), recovered (bright blue), remained on renal replacement therapy (dark blue) or reached the end of follow-up over time (yellow). (B) Cumulative incidence plots of time to initiation of renal replacement therapy from: Symptom onset (red), hospital admission (blue), intubation (yellow) and ICU admission (black).

**Fig 2:**
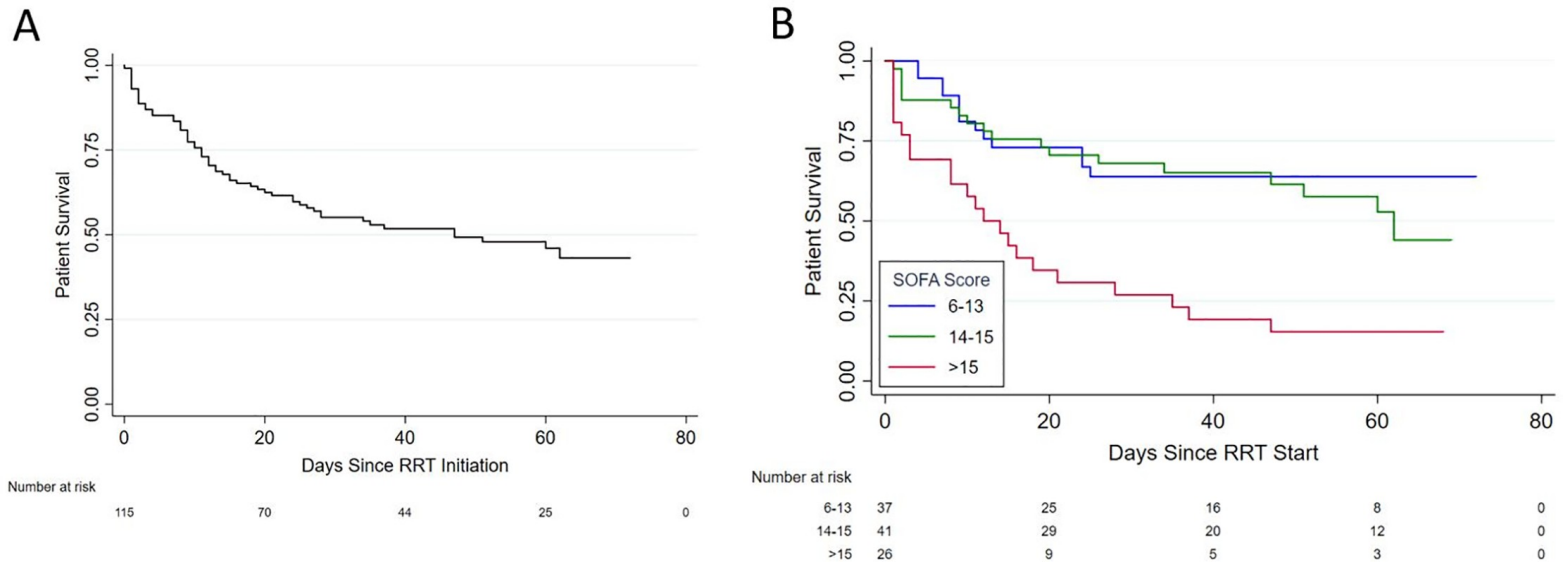
Patient survival curves. (A) Kaplan Meier curve for patient survival over time from the time of initiation of renal replacement therapy for all patients and (B) stratified by tertiles of SOFA scores.

During the study period a total of 510 patients with COVID19 were admitted to the ICU, among whom 115 (23%) received RRT for AKI (Table 1) and were followed for a median of 30 days (IQR: 11–58) from RRT initiation (Table 2). Of the patients alive at the end of follow-up, the median duration of follow-up was 57 days for patients who recovered and 54 days for patients with persistent RRT indications (Table 2, Fig 1A). By the end of our follow-up period, 59 (51%) patients died (survival probability at 60 days: 0.46 [95% CI: 0.36–0.56]), 47 patients (41%) had evidence of kidney recovery, and the remaining 9 patients (8%) had continued indications for RRT (Table 1, Figs 1A & 2A, S1 Fig). Of these 9 patients with persistent RRT indications, 7 remained hospitalized at the end of the study follow-up period, while 1 had chosen to withdraw RRT support and was still hospitalized, and 2 were discharged with dialysis-dependent AKI. Of the 47 patients who survived and had kidney recovery, the median Cr and eGFR by CKD-EPI at the time of discharge or end of follow-up were 1.25mg/dL [0.86–2.13] and 61mL/min/1.73m2 [29–92], respectively. At the height of the surge, the peak prevalence of AKI requiring RRT in the ICU was 29% (67/231).

**Table 1:** Baseline demographics and clinical characteristics of the cohort stratified by patient status at the end of follow-up.

	All	Died	Alive	
			Recovered	RRT Still Indicated
	**n = 115**	**n = 59**	**n = 47**	**n = 9**
**Total**	115 (100)	59 (51)	47 (41)	9 (8)
**Demographics**				
** Age** (years)	63 (53–71)	65 (56–72)	60 (53–64)	61 (47–75)
** Sex**				
Female	31 (27)	14 (24)	14 (30)	3 (33)
Male	84 (73)	45 (76)	33 (70)	6 (67)
** Race**				
Black	37 (32)	16 (27)	18 (38)	3 (33)
White	26 (23)	13 (22)	10 (21)	3 (33)
Asian	5 (4)	2 (3)	3 (6)	0 (0)
Multi-racial	27 (23)	16 (27)	9 (19)	2 (22)
Not reported	20 (17)	12 (20)	7 (15)	1 (11)
** Ethnicity**				
Hispanic/Latino	60 (52)	36 (61)	17 (36)	7 (78)
Not Hispanic/Latino	48 (42)	21 (36)	25 (53)	2 (22)
Not reported	7 (6)	2 (3)	5 (11)	0 (0)
**Past Medical History**				
** BMI**^a^	31.0 (25.8–35.2)	30.3 (24.9–35.1)	32.5 (27.2–36.4)	26.9 (25.7–31.2)
≤30	52 (46)	28 (48)	19 (40)	5 (56)
>30	62 (54)	30 (52)	28 (60)	4 (44)
** Smoking**				
Never	51 (44)	23 (39)	25 (53)	3 (33)
Active	9 (8)	5 (8)	4 (9)	0 (0)
Former	20 (17)	13 (22)	6 (13)	1 (11)
Unknown	35 (30)	18 (31)	12 (26)	5 (56)
** Baseline Cr**^b^	1.2 (0.9–1.7)	1.2 (1.0–1.9)	1.2 (0.9–1.5)	1.5 (0.8–2.2)
** CKD**				
Stage 3	17 (15)	9 (15)	5 (11)	3 (33)
Stage 4	5 (4)	4 (7)	1 (2)	0 (0)
Stage 5	4 (3)	1 (2)	2 (4)	1 (11)
Unspecified stage	6 (5)	1 (2)	4 (9)	1 (11)
** HTN**	81 (70)	45 (76)	29 (62)	7 (78)
** DM**	57 (50)	29 (49)	21 (45)	7 (78)
** CAD**	12 (10)	8 (14)	2 (4)	2 (22)
** CHF**	9 (8)	4 (7)	4 (9)	1 (11)
** CVA**	6 (5)	1 (2)	4 (9)	1 (11)
** COPD**	8 (7)	7 (12)	1 (2)	0 (0)
** Asthma**	11 (10)	5 (8)	6 (13)	0 (0)
** HIV**	5 (4)	3 (5)	1 (2)	1 (11)
** Active Malignancy**	7 (6)	3 (5)	4 (9)	0 (0)
** Transplant**	6 (5)	2 (3)	4 (9)	0 (0)
**Outpatient Medications**				
** NSAIDs**	12 (10)	6 (10)	5 (11)	1 (11)
** RAASi**	46 (40)	29 (49)	14 (30)	3 (33)
ACEi	29 (25)	21 (36)	7 (15)	1 (11)
ARB	17 (15)	8 (14)	7 (15)	2 (22)
** Steroid**	3 (3)	1 (2)	2 (4)	0 (0)
**Immunosuppressants**	7 (6)	4 (7)	3 (6)	0 (0)
** Azithromycin**	8 (7)	3 (5)	5 (11)	0 (0)
**Hydroxychloroquine**	6 (5)	2 (3)	4 (9)	0 (0)

^a^ n = 114 (1 patient without height to calculate BMI).

^b^ among 34 patients with a baseline creatinine value available prior to hospital presentation.

RRT, renal replacement therapy; BMI, body-mass index; Cr, creatinine; CKD, chronic kidney disease; HTN, hypertension; DM, diabetes mellitis; CAD, coronary artery disease; CHF, congestive heart failure; CVA, cerebrovascular accident; COPD, chronic obstructive pulmonary disease; HIV, human immunodeficiency virus; NSAIDs, non-steroidal anti-inflammatory drugs; RAASi, renin-angiotensin-aldosteron system inhibition; ACEi, angiotensin-converting enzyme inhibitor; ARB, angiotensin receptor blocker.

**Table 2:** Illness severity and clinical outcomes of the cohort stratified by patient status at the end of follow-up.

		All	Died	Alive	
				Recovered	RRT Still Indicated
	**n**	**115**	**59**	**47**	**9**
**Illness Severity**					
SOFA					
at ICU Admission	111	14 (12–15)	14 (12–16)	13 (11–14)	14 (13–15)
at RRT Initiation	104	14 (13–16)	14 (14–17)	14 (12–15)	14 (13–15)
Mechanical Ventilation					
Any		114 (99)	58 (98)	47 (100)	9 (100)
at RRT Initiation		109 (95)	55 (93)	47 (100)	7 (78)
Lowest PaO_2_:FiO_2_ Ratio					
at ICU Admission	112	137 (87–201)	125 (83–171)	145 (91–204)	163 (100–258)
at RRT Initiation	108	153 (106–227)	126 (93–204)	197 (114–235)	180 (131–231)
Vasopressors at RRT initiation		97 (84)	52 (88)	38 (81)	7 (78)
Urine output at RRT initiation (L)^a^		0.30 (0.03–0.73)	0.12 (0.00–0.65)	0.48 (0.16–1.00)	0.24 (0.00–0.90)
**Initial Indication for RRT**					
Hypoxia or Overload		20 (17)	7 (12)	11 (23)	2 (22)
Hyperkalemia		8 (7)	5 (8)	2 (4)	1 (11)
Acidemia		8 (7)	4 (7)	4 (9)	0 (0)
Uremia		5 (4)	3 (5)	2 (4)	0 (0)
Multiple		58 (50)	31 (53)	21 (45)	6 (67)
Not specified		16 (14)	9 (15)	7 (15)	0 (0)
**Initial RRT Modality**					
CRRT		105 (91)	53 (90)	45 (96)	7 (78)
HD		10 (9)	6 (10)	2 (4)	2 (22)
**RRT Modality through LOS**					
CRRT only		57 (50)	44 (75)	13 (28)	0 (0)
HD only		2 (2)	1 (2)	1 (2)	0 (0)
CRRT + HD		52 (45)	11 (19)	33 (70)	8 (89)
CRRT + PD		2 (2)	2 (3)	0 (0)	0 (0)
CRRT + HD + PD		2 (2)	1 (2)	0 (0)	1 (11)
**Time from Symptom Onset** (days)					
To Presentation	94	6 (3–9)	7 (3–11)	6 (4–9)	7 (6–15)
To RRT Start	94	12 (9–16)	12 (8–16)	12 (9–16)	16 (10–28)
**Admission to RRT Initiation** (days)					
Hospital Admission		5 (2–8)	4 (1–7)	5 (3–9)	4 (2–5)
ICU Admission		2 (1–5)	2 (0–3)	2 (1–6)	2 (1–2)
**Duration of Therapy** (days)					
RRT		19 (8–35)	11 (3–21)	25 (16–37)	54 (43–62)
Mechanical Ventilation^b^		22 (13–55)	15 (9–24)	48 (20–65)	67 (55–67)
**Length of Stay** (days)					
Hospitalization		36 (19–63)	19 (10–29)	63 (39–70)	67 (55–72)
ICU Admission		25 (13–44)	15 (6–26)	41 (23–57)	50 (32–61)
**Overlap of RRT**					
% of Hospital LOS on RRT		57 (40–81)	65 (43–85)	47 (32–63)	91 (81–97)
% of ICU LOS on RRT		77 (52–93)	80 (58–98)	70 (48–84)	93 (83–96)
**Median follow-up time** (days)		29 (11–58)	11 (3–24)	57 (32–62)	54 (50–61)
**Patient RRT days**		2542	867	1219	456
**Patient ICU days**		3461	1130	1911	420

^a^ urine output for the 24 hours preceding RRT initiation.

^b^ Only days since the patient was admitted were counted. 11 patients were intubated elsewhere prior to CUIMC admission.

SOFA, Sequential Organ Function Assessment; RRT, renal replacement therapy; PaO2, partial pressure of oxygen in arterial blood; FiO2, inhaled oxygen concentration as %; LOS, length of stay; CRRT, continuous renal replacement therapy; HD, hemodialysis; PD, peritoneal dialysis.

The demographics of our cohort are outlined in Table 1. The median age was 63 years (IQR 53–71), 73% were male, 32% were black, 23% were white and approximately half (52%) identified as Hispanic. The median body mass index (BMI) of our cohort was 31 kg/m^2^ (IQR 26–35, n = 114) with 55% classified as obese (BMI > 30; Table 1). The most common comorbidities included hypertension (70%), diabetes (50%), chronic kidney disease stages 3–5 (25%) and asthma or chronic obstructive lung disease (COPD; 17%). Outpatient medication use included 25% of patients on angiotensin converting enzyme inhibitors (ACEi), 15% on angiotensin receptor blockers (ARB), 10% on non-steroidal anti-inflammatory drugs (NSAIDs), 6% on immunosuppressants, 7% on azithromycin, and 5% on hydroxychloroquine (Table 1).

The vast majority of our patients presented with symptoms characteristic of COVID-19 infection (n = 111, 97%; S2 Table). The median time from symptom onset to presentation was 6 days (IQR 3–9; n = 94 patients with available date of symptom onset) while the median time to RRT initiation was: 12 days (IQR 9–16) from symptom onset (n = 94 patients with a known date of symptom onset), 5 days (IQR 2–8) from hospital admission, and 2 days (IQR 1–5) from ICU admission (Table 2, Fig 1B).

Consistent with hospital protocols at the time 98% of patients received treatment targeting SARS-CoV-2 including hydroxychloroquine (89%) and azithromycin (83%; S2 Table) and 99% percent of our cohort required mechanical ventilation with a median nadir PaO_2_:FiO_2_ ratio <200 at ICU admission (Table 2). The median SOFA score at ICU admission and RRT initiation was 14 (Table 2). The majority of patients were on mechanical ventilation (99%) and required vasopressor agents (84%) at the time of RRT initiation. CRRT was the initial RRT modality for 91% of patients, and the majority were oligo-anuric (24-hour urine output <0.5L) in the 24 hours leading up to RRT initiation and had multiple indications for RRT (Table 2).

Fifty-one patients (44%) had a urinalysis performed during their first 24h in the ICU, of which 50 (98%) had evidence of proteinuria and 35 (69%) had evidence of hematuria (S3 Table). While markers of inflammation, anaerobic metabolism and degradation products/cell turnover were elevated in all patients, they were highest in the group that died (S3 and S4 Tables).

Variables associated with increased mortality in univariate analysis included increasing age, COPD, outpatient ACEi use, urine output in the 24 hours preceding RRT initiation and higher SOFA scores at the time of RRT initiation (Table 3). Patients with a SOFA score 6–13 experienced similar mortality as those patients with a score of 14–15, but significantly better survival than those with a score > 15 (Table 3, Fig 2B). With the exception of COPD, on univariate analysis comorbidities (including hypertension and diabetes) were not associated with increased mortality, nor were markers of inflammation (Table 3). In a multivariable Cox proportional hazards model that included age, sex, race and other significant clinical factors from the univariate analysis, we found that CAD and COPD were both associated with increased mortality (HR 3.99 [95% CI 1.46–10.90] and 3.10 [95% CI 1.25–7.66] respectively), as was the prior use of ACEi (HR 2.33 [95% CI 1.21–4.47]) and SOFA scores > 15 (HR 3.46 [95% CI 1.65–7.25]; Table 3). Age and sex were not associated with increased mortality in this model. Given the small proportion of patients with coronary artery disease (10%) and COPD (7%), we also considered a second multivariable model that did not include comorbidities. In the second model, associations with mortality from the primary model remained unchanged.

**Table 3:** Survival analysis with univariate and multivariable models showing hazard ratios for death.

			Unadjusted			Adjusted 1			Adjusted 2		
	per	n^a^	HR	95% CI	p	HR	95% CI	p	HR	95% CI	p-value
**Demographics**											
Age	1 year		1.03	(1.00, 1.05)	0.023	1.01	(0.98, 1.03)	0.493	1.02	(0.99, 1.04)	0.128
Sex											
Female			reference			reference			reference		
Male			1.20	(0.66, 2.18)	0.555	0.96	(0.50, 1.85)	0.907	0.92	(0.47, 1.77)	0.793
Race											
Black			0.95	(0.46, 1.98)	0.893	1.54	(0.66, 3.63)	0.320	0.96	(0.44, 2.10)	0.916
White			reference			reference			reference		
Asian			0.92	(0.21, 4.10)	0.918	1.48	(0.29, 7.59)	0.642	0.77	(0.16, 3.64)	0.738
Multi-racial			1.44	(0.69, 3.01)	0.329	1.63	(0.67, 3.94)	0.282	1.13	(0.50, 2.59)	0.764
Not reported			1.51	(0.69, 3.32)	0.302	1.26	(0.48, 3.30)	0.633	0.82	(0.33, 2.01)	0.662
Ethnicity											
Hispanic/Latino			1.51	(0.88, 2.58)	0.137						
Not Hispanic/Latino			reference								
Not reported			0.68	(0.16, 2.90)	0.600						
**Past Medical History**											
BMI	1 unit	114	1.00	(0.96, 1.03)	0.812						
Smoking											
Never			reference								
Active			1.48	(0.56, 3.91)	0.424						
Former			1.64	(0.83, 3.24)	0.156						
Unknown			1.15	(0.62, 2.13)	0.655						
CKD			0.85	(0.48, 1.50)	0.567						
HTN			1.56	(0.85, 2.84)	0.149						
DM			1.11	(0.67, 1.85)	0.687						
CAD			2.04	(0.97, 4.31)	0.061	3.99	(1.46, 10.90)	0.007			
CHF			0.87	(0.31, 2.39)	0.781						
CVA			0.28	(0.04, 1.99)	0.202						
COPD			2.88	(1.30, 6.38)	0.009	3.10	(1.25, 7.66)	0.014			
Asthma			0.85	(0.34, 2.13)	0.728						
HIV			1.41	(0.44, 4.53)	0.559						
Malignancy			0.61	(0.19, 1.96)	0.407						
Transplant			0.50	(0.12, 2.05)	0.335						
**Outpatient Medications**											
NSAIDs			0.96	(0.41, 2.24)	0.928						
ACEi			2.13	(1.25, 3.64)	0.006	2.33	(1.21, 4.47)	0.011	2.29	(1.18, 4.43)	0.014
ARB			1.13	(0.53, 2.37)	0.757	1.62	(0.63, 4.21)	0.320	2.03	(0.81, 5.09)	0.131
Steroid			0.57	(0.08, 4.14)	0.579						
Immunosuppressants			0.89	(0.32, 2.46)	0.823						
Azithromycin			0.62	(0.19, 1.98)	0.420						
**Illness Severity**											
SOFA Score at RRT start											
6–13			reference			reference			reference		
14–15			1.22	(0.60, 2.49)	0.585	0.94	(0.43, 2.06)	0.883	0.99	(0.45, 2.13)	0.970
>15			3.54	(1.78, 7.05)	< 0.001	3.46	(1.65, 7.25)	0.001	3.19	(1.52, 6.71)	0.002
Missing			1.75	(0.66, 4.60)	0.258	1.25	(0.44, 3.59)	0.674	1.34	(0.48, 3.70)	0.574
SOFA at RRT start	1 unit	104	1.25	(1.09, 1.44)	0.001						
**Labs/Additional**											
ESR, mm/hr	10 units	64	1.05	(0.93, 1.18)	0.462						
CRP, mg/L	10 units	67	0.99	(0.95, 1.03)	0.571						
IL-6, pg/mL	10 units	39	1.01	(0.97, 1.05)	0.734						
D-dimer, mg/L	1 units	68	1.01	(0.96, 1.06)	0.814						
Ferritin, ng/mL	100 units	61	1.00	(0.99, 1.01)	0.944						
Urine Output^b^	1 units		0.51	(0.29, 0.92)	0.024	0.58	(0.30, 1.12)	0.102	0.60	(0.33, 1.11)	0.104
Anticoagulation	yes		0.87	(0.31, 2.43)	0.795						

^a^ when n<117.

^b^ urine output measured for the 24 hours preceding RRT initiation.

BMI, body-mass index; CKD, chronic kidney disease; HTN, hypertension; DM, diabetes mellitis; CAD, coronary artery disease; CHF, congestive heart failure; CVA, cerebrovascular accident; COPD, chronic obstructive pulmonary disease; HIV, human immunodeficiency virus; NSAIDs, non-steroidal anti-inflammatory drugs; RAASi, renin-angiotensin-aldosteron system inhibition; ACEi, angiotensin-converting enzyme inhibitor; ARB, angiotensin receptor blocker; SOFA, sequential organ function assessment; RRT, renal replacement therapy; ESR, erythrocyte sedimentation rate; CRP, C-reactive protein; IL-6, interleukin-6; UOP, urine output.

The median length of stay (LOS) was 36 days (IQR 19–63) for hospital admission and 25 days (IQR 13–44) for ICU admission. The median duration of RRT for the entire cohort was 19 days (IQR 8–35), representing a total of 2542 patient RRT days. Days on RRT represented 57% of total hospital days and 77% of ICU days (Table 2).

## Discussion

Our analysis provides a detailed characterization of a cohort of 115 critically ill patients with COVID-19 who develop severe AKI requiring RRT, representing almost one quarter of all patients admitted to the ICU at a large, urban, academic, quaternary medical center during the COVID pandemic. Importantly, although approximately half of these patients died, the majority (84%) of those who survived had sufficient recovery of kidney function to allow RRT cessation. This detailed assessment with extended follow up provides important new understanding of the prognosis for patients with severe COVID-19 who require both mechanical ventilation for ARDS and RRT for severe AKI.

The overwhelming majority of the patients with COVID-19 admitted to the ICU at our institution required invasive mechanical ventilation and most required pressor support, reflective of the high thresholds for ICU admission during the surge. As a result, SOFA scores for our cohort are significantly higher than severity scores from other large ICU cohort studies that included small proportion of individuals with COVID-19 needing mechanical ventilation (42–82%) or vasopressors (30–67%) [15,17–19,21].

Consistent with prior reports, increasing age was associated with increased mortality in unadjusted analyses. In addition, we noted increased mortality associated with the presence of comorbidities of CAD and COPD, the prior use of ACEi and increasing SOFA scores (Table 3). Notably we did not find an association with hypertension, diabetes, or chronic kidney disease–which may be a function of the high prevalence of these in our cohort. Similarly, over half the cohort was obese but obesity was not associated with increased mortality in this cohort. Traditional markers of inflammation at the time of ICU admission were not associated with mortality. The association of the prior use of ACEi and higher SOFA scores > 15 (Table 3) persisted in our multivariable analysis while the associations with age and urine output did not. The exact role renin-angiotensin-aldosterone system agents have on both infection rates and mortality remains controversial, and the consequences of their subsequent discontinuation in the hospital setting in COVID-19 remain unclear. [24–26], this has not been seen consistently in other cohorts and likely requires further study [27,28]. Notably, despite lower severity of illness, prior ICU cohorts reported mortality rates of 75–89% for patients with COVID-19 requiring RRT with shorter follow up [12,17,19]. The reasons for the differences in mortality are unclear. The majority of our patient cohort was oligoanuric at the time of initiation of RRT and most patients were treated with CRRT soon after ICU admission. Given prior evidence on the role of the timing of dialysis initiation, it is unlikely that the prompt initiation of RRT was protective [29–31].

While the exact mechanisms of kidney injury in patients with COVID-19 remain to be fully understood [32], there are additional concerns about AKI that results from associated hemodynamic instability and/or volume depletion occurring in the management of ARDS. As a result, different clinical phenotypes may be associated with divergent prognoses, but accurately differentiating these phenotypes is challenging. Heterogeneous mortality rates across different ICU cohorts may result from varied thresholds for the initiation of RRT and likely reflect resource constraints and the existing clinical practice or from differences in illness severity that are not adequately captured by the SOFA score [33,34].

Our analysis provides a detailed characterization of patients with severe COVID-19 infection on mechanical ventilation, vasopressors and in need of RRT for severe AKI. Although the mortality rate for patients in our cohort were high, the high rates of kidney recovery in those patients who survived is particularly encouraging. The impact on the overall burden of CKD in survivors of COVID-19 associated AKI remains to be determined, but the immediate burden of dialysis-dependent AKI appears to be small and the overwhelming majority of those who have kidney recovery will experience this prior to discharge, consistent with prior reports on the recovery of dialysis-dependent AKI [35]. These findings are urgently needed to help clinicians make more reliable prognostic estimates for individual patients who develop COVID-19 associated AKI and should inform the shared decision making discussions for critically ill patients and their families. On a systems level, our findings inform the need for hospitals to consider and plan for RRT needs in the midst of the pandemic, and for healthcare systems to consider the long-term implications of an increase in dialysis-dependent AKI that are likely to outlast the pandemic.

While our description details the clinical outcomes of critically ill patients requiring RRT, our analysis may not be generalizable to critically ill patients at other centers. Given that 95% of our cohort was mechanically ventilated and 84% required vasopressors at the time of RRT initiation, our results may not be directly applicable in ICUs with lower rates of mechanical ventilation and vasopressor use. In addition, despite our lengthy follow-up (median >54 days from RRT initiation for survivors), a small proportion of patients were still hospitalized, and their final disposition is therefore indeterminable. Finally, many patients presenting with COVID-19 had no prior medical records available, creating a challenge when trying to determine their baseline kidney function. While some cohorts have had as few as 15% of patients with a prior SCr [14], our cohort was marginally better with 30% of patients having a pre-hospitalization SCr available for baseline SCr assessment. This in turn may result in the underestimation of the burden of pre-existing chronic kidney disease in our cohort as well as its influence on the overall prognosis.

In conclusion, our study demonstrates a high incidence (23%) and peak prevalence (29%) of severe AKI requiringRRT among critically ill patients at the epicenter of the COVID-19 pandemic in the United States, which highlights this important and often overlooked need during disaster resource planning. While we identify the grim prognosis of COVID-19 associated severe AKI occurring in patients requiring mechanical ventilation with a 51% mortality rate, our results also demonstrate a high rate of recovery of kidney function among survivors (84%). These results fill in an important gap in our understanding of the prognosis of the sickest patients and will inform shared decision-making discussions between patients, their family members, and providers regarding life-sustaining interventions in the face of COVID-19.

## Supporting information

S1 Fig Flowchart demonstrating patient accounting by outcomes. (TIF)

S1 Table Transformations of categorical lab values. (TIF)

S2 Table Details of patients’ COVID-19 presentation and treatment. (TIF)

S3 Table Clinical laboratory values at the time of ICU admission and at the time of RRT initiation stratified by patient status at the end of follow-up. (TIF)

S4 Table Minimum and maximum laboratory values throughout admission. (TIF)

## References

[1] Liu KD, Glidden DV, Eisner MD, Parsons PE, Ware LB, Wheeler A, et al Predictive and pathogenetic value of plasma biomarkers for acute kidney injury in patients with acute lung injury. Crit Care Med. 2007;35(12):2755–61. . 18074478 PMC3293249

[2] Darmon M, Clec'h C, Adrie C, Argaud L, Allaouchiche B, Azoulay E, et al Acute respiratory distress syndrome and risk of AKI among critically ill patients. Clin J Am Soc Nephrol. 2014;9(8):1347–53. Epub 05/29. 10.2215/CJN.08300813 . 24875195 PMC4123396

[3] Comparison of Two Fluid-Management Strategies in Acute Lung Injury. New England Journal of Medicine. 2006;354(24):2564–75. 10.1056/NEJMoa062200 . 16714767

[4] Cooke CR, Kahn JM, Caldwell E, Okamoto VN, Heckbert SR, Hudson LD, et al Predictors of hospital mortality in a population-based cohort of patients with acute lung injury*. Read Online: Critical Care Medicine | Society of Critical Care Medicine. 2008;36(5):1412–20. 10.1097/CCM.0b013e318170a375 00003246-200805000-00003. 18434894

[5] Cheng Y, Luo R, Wang K, Zhang M, Wang Z, Dong L, et al Kidney disease is associated with in-hospital death of patients with COVID-19. Kidney Int. 2020;97(5):829–38. Epub 2020/04/06. 10.1016/j.kint.2020.03.005 32247631 PMC7110296

[6] Robbins-Juarez SY, Qian L, King KL, Stevens JS, Husain SA, Radhakrishnan J, et al Outcomes for Patients With COVID-19 and Acute Kidney Injury: A Systematic Review and Meta-Analysis. Kidney Int Rep. 2020;5(8):1149–60. Epub 2020/08/11. 10.1016/j.ekir.2020.06.013 32775814 PMC7314696

[7] Bhatraju PK, Ghassemieh BJ, Nichols M, Kim R, Jerome KR, Nalla AK, et al Covid-19 in Critically Ill Patients in the Seattle Region—Case Series. N Engl J Med. 2020 Epub 2020/04/01. 10.1056/NEJMoa2004500 32227758 PMC7143164

[8] Grasselli G, Pesenti A, Cecconi M. Critical Care Utilization for the COVID-19 Outbreak in Lombardy, Italy: Early Experience and Forecast During an Emergency Response. JAMA. 2020 Epub 2020/03/14. 10.1001/jama.2020.4031 . 32167538

[9] Wu C, Chen X, Cai Y, Xia J, Zhou X, Xu S, et al Risk Factors Associated With Acute Respiratory Distress Syndrome and Death in Patients With Coronavirus Disease 2019 Pneumonia in Wuhan, China. JAMA Intern Med. 2020 Epub 2020/03/14. 10.1001/jamainternmed.2020.0994 32167524 PMC7070509

[10] Guan WJ, Ni ZY, Hu Y, Liang WH, Ou CQ, He JX, et al Clinical Characteristics of Coronavirus Disease 2019 in China. N Engl J Med. 2020;382(18):1708–20. Epub 2020/02/29. 10.1056/NEJMoa2002032 32109013 PMC7092819

[11] Goyal P, Choi JJ, Pinheiro LC, Schenck EJ, Chen R, Jabri A, et al Clinical Characteristics of Covid-19 in New York City. N Engl J Med. 2020;382(24):2372–4. Epub 2020/04/18. 10.1056/NEJMc2010419 32302078 PMC7182018

[12] Pei G, Zhang Z, Peng J, Liu L, Zhang C, Yu C, et al Renal Involvement and Early Prognosis in Patients with COVID-19 Pneumonia. J Am Soc Nephrol. 2020 Epub 2020/04/30. 10.1681/ASN.2020030276 . 32345702 PMC7269350

[13] Richardson S, Hirsch JS, Narasimhan M, Crawford JM, McGinn T, Davidson KW, et al Presenting Characteristics, Comorbidities, and Outcomes Among 5700 Patients Hospitalized With COVID-19 in the New York City Area. JAMA. 2020 Epub 2020/04/23. 10.1001/jama.2020.6775 32320003 PMC7177629

[14] Hirsch JS, Ng JH, Ross DW, Sharma P, Shah HH, Barnett RL, et al Acute kidney injury in patients hospitalized with COVID-19. Kidney Int. 2020;98(1):209–18. Epub 2020/05/18. 10.1016/j.kint.2020.05.006 32416116 PMC7229463

[15] Wang D, Hu B, Hu C, Zhu F, Liu X, Zhang J, et al Clinical Characteristics of 138 Hospitalized Patients With 2019 Novel Coronavirus-Infected Pneumonia in Wuhan, China. JAMA. 2020 Epub 2020/02/08. 10.1001/jama.2020.1585 32031570 PMC7042881

[16] Huang C, Wang Y, Li X, Ren L, Zhao J, Hu Y, et al Clinical features of patients infected with 2019 novel coronavirus in Wuhan, China. The Lancet. 2020;395(10223):497–506. 10.1016/s0140-6736(20)30183-5 31986264 PMC7159299

[17] Yang X, Yu Y, Xu J, Shu H, Xia Ja, Liu H, et al Clinical course and outcomes of critically ill patients with SARS-CoV-2 pneumonia in Wuhan, China: a single-centered, retrospective, observational study. Lancet Respir Med. 2020:S2213-600(20)30079-5. 10.1016/S2213-2600(20)30079-5 . 32105632 PMC7102538

[18] Arentz M, Yim E, Klaff L, Lokhandwala S, Riedo FX, Chong M, et al Characteristics and Outcomes of 21 Critically Ill Patients With COVID-19 in Washington State. JAMA. 2020 10.1001/jama.2020.4326 32191259 PMC7082763

[19] Intensive Care National Audit & Research Center [Website]. https://www.icnarc.org/Our-Audit/Audits/Cmp/Reports2020 [updated May 1, 2020; cited 2020 May 1]. Available from: https://www.icnarc.org/Our-Audit/Audits/Cmp/Reports.

[20] Argenziano MG, Bruce SL, Slater CL, Tiao JR, Baldwin MR, Barr RG, et al Characterization and clinical course of 1000 patients with coronavirus disease 2019 in New York: retrospective case series. BMJ. 2020;369:m1996 Epub 2020/05/31. 10.1136/bmj.m1996 www.icmje.org/coi_disclosure.pdf and declare: no support from any organization for the submitted work; no competing interests with regards to the submitted work; MMS reports grants from Amgen, outside the submitted work; JJC reports personal fees from Allergan, outside the submitted work; RGB reports grants from Alpha1 Foundation and COPD Foundation, outside the submitted work; GH reports grants from Janssen Research, outside the submitted work; the remaining authors have nothing to disclose. 32471884 PMC7256651

[21] Cummings MJ, Baldwin MR, Abrams D, Jacobson SD, Meyer BJ, Balough EM, et al Epidemiology, clinical course, and outcomes of critically ill adults with COVID-19 in New York City: a prospective cohort study. Lancet. 2020;395(10239):1763–70. Epub 2020/05/23. 10.1016/S0140-6736(20)31189-2 32442528 PMC7237188

[22] Vincent JL, Moreno R, Takala J, Willatts S, De Mendonca A, Bruining H, et al The SOFA (Sepsis-related Organ Failure Assessment) score to describe organ dysfunction/failure. On behalf of the Working Group on Sepsis-Related Problems of the European Society of Intensive Care Medicine. Intensive Care Med. 1996;22(7):707–10. Epub 1996/07/01. 10.1007/BF01709751 . 8844239

[23] Vasilevskis EE, Pandharipande PP, Graves AJ, Shintani A, Tsuruta R, Ely EW, et al Validity of a Modified Sequential Organ Failure Assessment Score Using the Richmond Agitation-Sedation Scale. Crit Care Med. 2016;44(1):138–46. Epub 2015/10/13. 10.1097/CCM.0000000000001375 26457749 PMC4748963

[24] Vaduganathan M, Vardeny O, Michel T, McMurray JJV, Pfeffer MA, Solomon SD. Renin-Angiotensin-Aldosterone System Inhibitors in Patients with Covid-19. N Engl J Med. 2020;382(17):1653–9. Epub 2020/04/01. 10.1056/NEJMsr2005760 32227760 PMC7121452

[25] Reynolds HR, Adhikari S, Pulgarin C, Troxel AB, Iturrate E, Johnson SB, et al Renin-Angiotensin-Aldosterone System Inhibitors and Risk of Covid-19. N Engl J Med. 2020;382(25):2441–8. Epub 2020/05/02. 10.1056/NEJMoa2008975 32356628 PMC7206932

[26] Mancia G, Rea F, Ludergnani M, Apolone G, Corrao G. Renin-Angiotensin-Aldosterone System Blockers and the Risk of Covid-19. N Engl J Med. 2020;382(25):2431–40. Epub 2020/05/02. 10.1056/NEJMoa2006923 32356627 PMC7206933

[27] Chung MK, Karnik S, Saef J, Bergmann C, Barnard J, Lederman MM, et al SARS-CoV-2 and ACE2: The biology and clinical data settling the ARB and ACEI controversy. EBioMedicine. 2020;58:102907 Epub 2020/08/11. 10.1016/j.ebiom.2020.102907 32771682 PMC7415847

[28] Kalra A, Hawkins ES, Nowacki AS, Jain V, Milinovich A, Saef J, et al Angiotensin-Converting Enzyme Inhibitors Versus Angiotensin II Receptor Blockers: A Comparison of Outcomes in Patients With COVID-19. Circ Cardiovasc Qual Outcomes. 2020;13(10):e007115 Epub 2020/08/29. 10.1161/CIRCOUTCOMES.120.007115 32856462 PMC7578112

[29] Barbar SD, Clere-Jehl R, Bourredjem A, Hernu R, Montini F, Bruyere R, et al Timing of Renal-Replacement Therapy in Patients with Acute Kidney Injury and Sepsis. N Engl J Med. 2018;379(15):1431–42. Epub 2018/10/12. 10.1056/NEJMoa1803213 . 30304656

[30] Zarbock A, Kellum JA, Schmidt C, Van Aken H, Wempe C, Pavenstadt H, et al Effect of Early vs Delayed Initiation of Renal Replacement Therapy on Mortality in Critically Ill Patients With Acute Kidney Injury: The ELAIN Randomized Clinical Trial. JAMA. 2016;315(20):2190–9. Epub 2016/05/23. 10.1001/jama.2016.5828 . 27209269

[31] Gaudry S, Hajage D, Schortgen F, Martin-Lefevre L, Pons B, Boulet E, et al Initiation Strategies for Renal-Replacement Therapy in the Intensive Care Unit. N Engl J Med. 2016;375(2):122–33. Epub 2016/05/18. 10.1056/NEJMoa1603017 . 27181456

[32] Puelles VG, Lutgehetmann M, Lindenmeyer MT, Sperhake JP, Wong MN, Allweiss L, et al Multiorgan and Renal Tropism of SARS-CoV-2. N Engl J Med. 2020 Epub 2020/05/14. 10.1056/NEJMc2011400 . 32402155 PMC7240771

[33] Cerda J, Mohan S, Garcia-Garcia G, Jha V, Samavedam S, Gowrishankar S, et al Acute Kidney Injury Recognition in Low- and Middle-Income Countries. Kidney Int Rep. 2017;2(4):530–43. Epub 2017/10/17. 10.1016/j.ekir.2017.04.009 29034358 PMC5637391

[34] Kher V, Srisawat N, Noiri E, Gharbi MB, Shetty MS, Yang L, et al Prevention and therapy of acute kidney injury in the developing world. Kidney international reports. 2017;2(4):544–58.

[35] Mohan S, Huff E, Wish J, Lilly M, Chen SC, McClellan WM, et al Recovery of renal function among ESRD patients in the US medicare program. PLoS One. 2013;8(12):e83447 Epub 2013/12/21. 10.1371/journal.pone.0083447 24358285 PMC3866227

