# Supplementary figures and images for: High rate of renal recovery in survivors of COVID-19 associated acute renal failure requiring renal replacement therapy
Source: PLoS One. 2020 Dec 28;15(12):e0244131. doi: 10.1371/journal.pone.0244131 (PMC7769434; doi:10.1371/journal.pone.0244131)

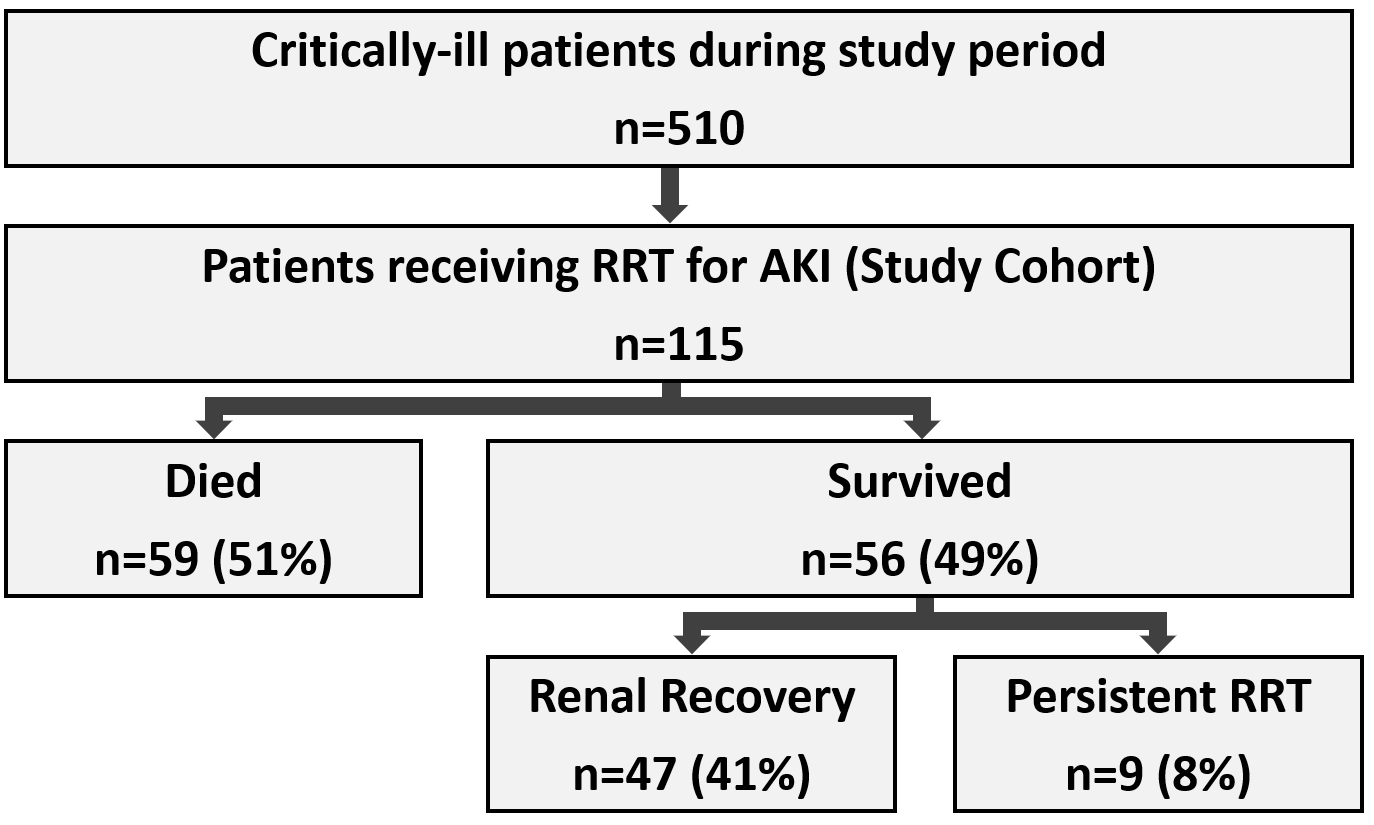

Supplement: S1 Fig — (TIF) [file pone.0244131.s001.tif]

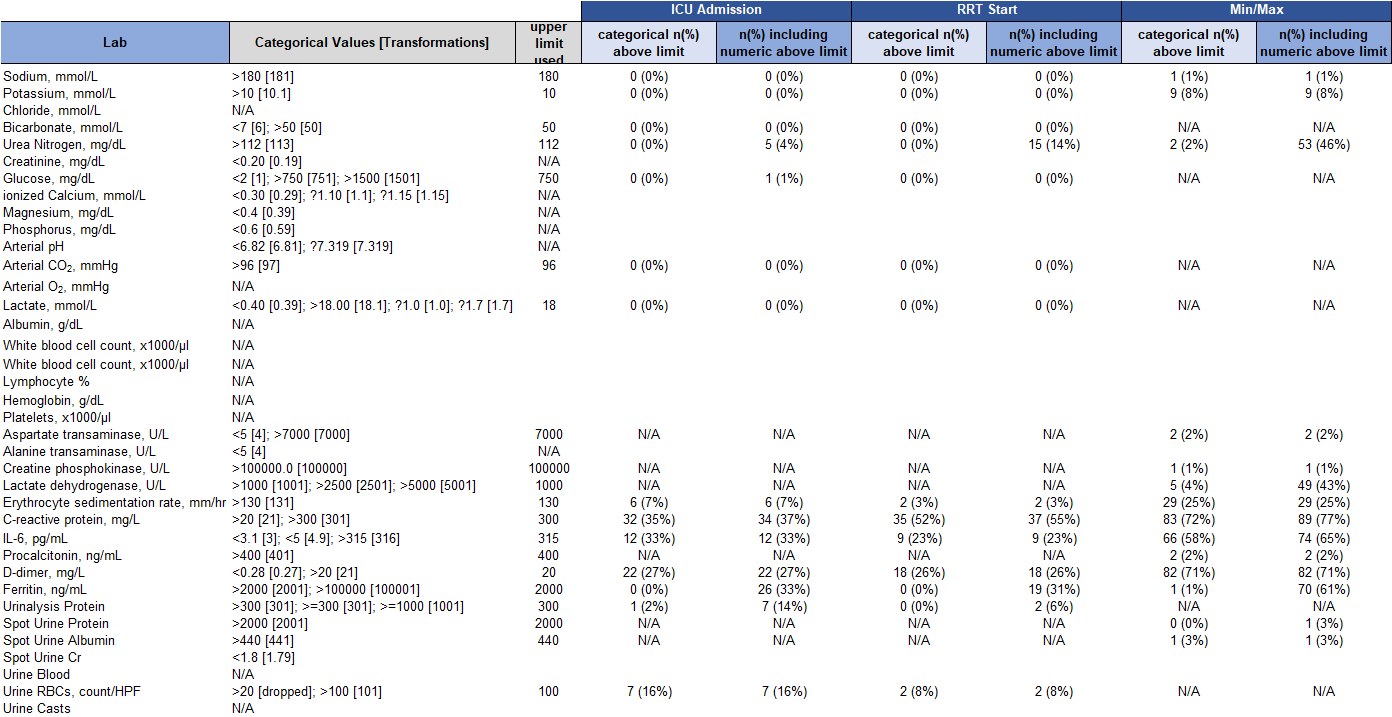

Supplement: S1 Table — (TIF) [file pone.0244131.s002.tif]

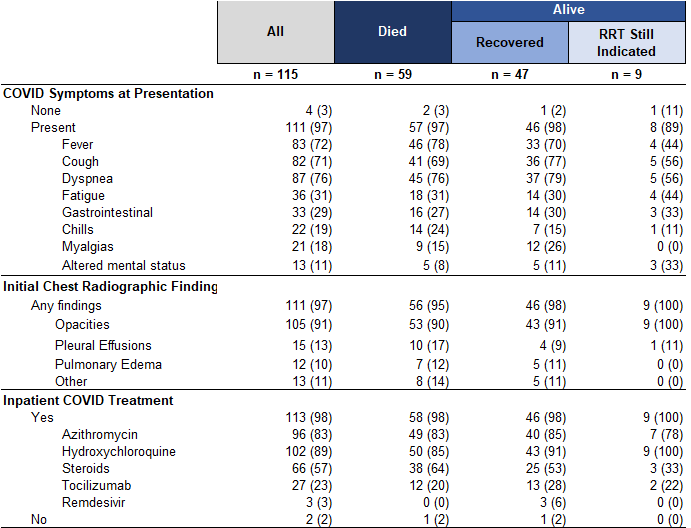

Supplement: S2 Table — (TIF) [file pone.0244131.s003.tif]

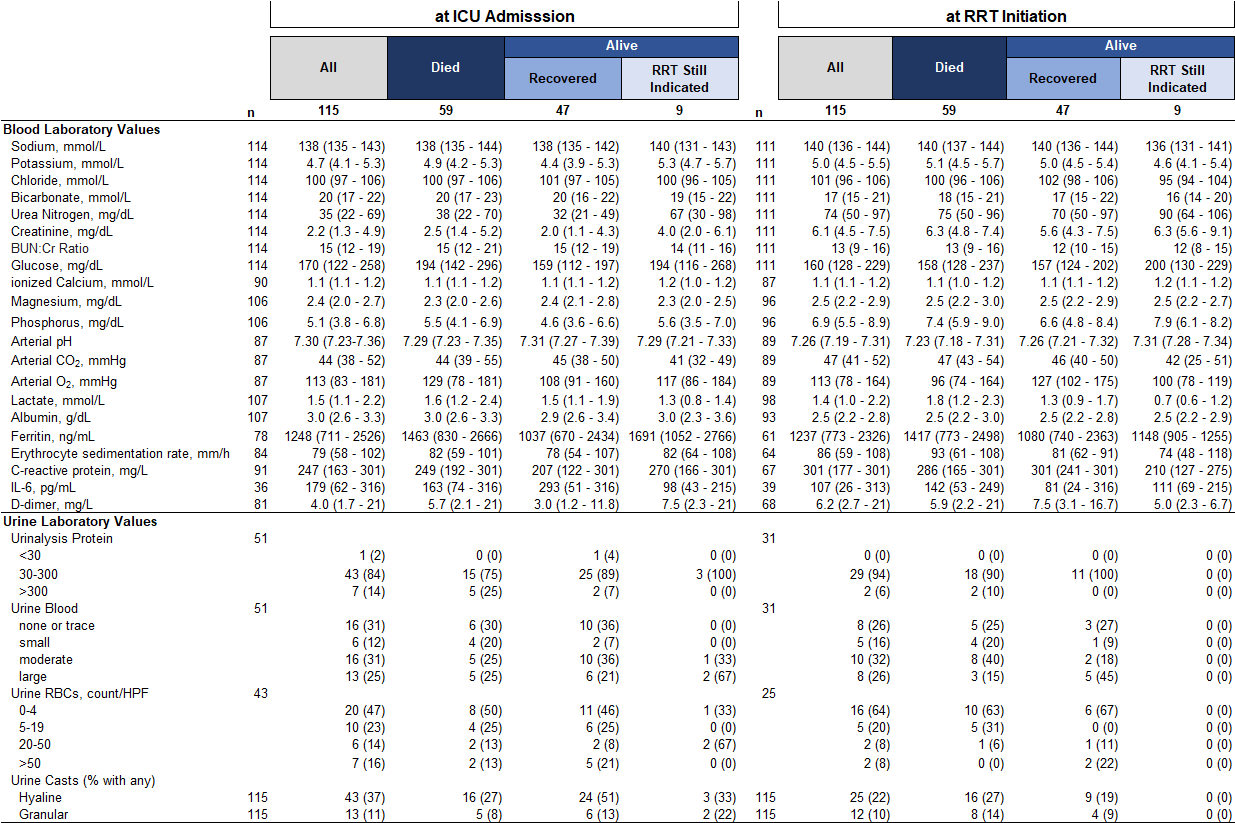

Supplement: S3 Table — (TIF) [file pone.0244131.s004.tif]

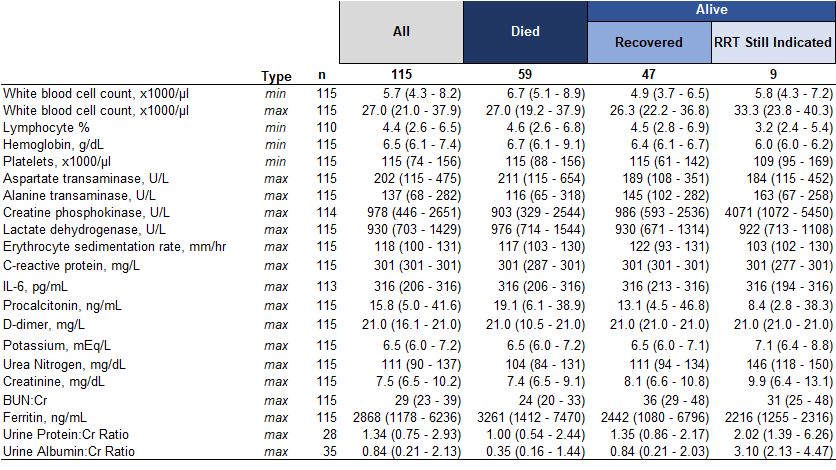

Supplement: S4 Table — (TIF) [file pone.0244131.s005.tif]
